# Supplementary material for: Experimental realisations of the fractional Schrödinger equation in the temporal domain
Source: Nat Commun. 2023 Jan 14;14:222. doi: 10.1038/s41467-023-35892-8 (PMC9840624; doi:10.1038/s41467-023-35892-8)
Supplement: Supplementary file 1 — Supplementary Information [file 41467_2023_35892_MOESM1_ESM.pdf]

Supplementary information: Experimental realisations of the fractional Schrödinger equation in the temporal domain

Shilong Liu[*]

Department of Physics, University of Ottawa,
25 Templeton Street, K1N 6N5, Ottawa, ON, Canada and
State Key Laboratory of Modern Optical Instrumentation,
College of Optical Science and Engineering,
Zhejiang University, Hangzhou, Zhejiang 310027, China

Yingwen Zhang and Ebrahim Karimi[†]

Department of Physics, University of Ottawa,
25 Templeton Street, K1N 6N5, Ottawa, ON, Canada and
National Research Council of Canada, 100 Sussex Drive, K1A 0R6, Ottawa, ON, Canada

Boris A. Malomed

Department of Physical Electronics, Faculty of Engineering,
and Center for Light-Matter Interaction, Tel Aviv University, Tel Aviv 69978, Israel and
Instituto de Alta Investigación, Universidad de Tarapacá, Casilla 7D, Arica, Chile

[*] dr.shilongliu@gmail.com

[†] ekarimi@uottawa.ca

Supplementary Note 1: The collinear Frequency-Resolved Optical Gating (FROG) system

The collinear Frequency-Resolved Optical Gating (FROG) system is used to reconstruct the amplitude and phase for an unknown temporal pulse. The extracted phase is then used to calibrate the SSI system. The input signal pulse with the central wavelength 816 nm is sent into a Michelson interferometer built around a PBS; the two outputs are then focused onto a thin BBO crystal (Type-II, e+o → e, Theta $= 41.9°$, radius $= 2500\ \mu$m, length $= 1000\ \mu$m), to perform the sum-frequency generation. The so-produced 408 nm pulse is then coupled into a spectrometer after filtering the pump beam with a low-pass filter.

To realize FROG reconstruction [1], the FROG trace is measured, by recording the spectrum $I(\omega)$ for different values of the relative temporal delay τ between the two pulses in the Michelson interferometer. For the type-II sum-frequency structure, the FROG trace is given by [2]

$$I_{\text{trace}}(\omega,\tau) = \left| \int_{-\infty}^{+\infty} E_H(t)E_V(t-\tau)\exp(-i\omega t)dt \right|^2, \tag{1}$$

where $E_H(t)$ and $E_V(t-\tau)$ are the two pulses with horizontal and vertical polarizations, respectively. Based on the FROG trace, we could reconstruct the amplitude and phase in the spectral or temporal domain, respectively, by using a generalized projection iterative algorithm [1].

Supplementary Figure 1 shows the recorded FROG trace and the reconstructed amplitude and phase in the temporal and spectral domains, respectively. The reconstructed spectral bandwidth for the reference and signal are 6.59 and 4.84 nm, respectively. The signal bandwidth is slightly narrower because of the presence of the tunable aperture inserted in the D-shaper system. The pulse durations obtained by reconstructions are 317 and 348 fs, respectively. Supplementary Figure 1(c) shows the case of a time-broadened signal pulse, where a second-order spectral phase is added by the spatial light modulator (SLM), simulating a pulse passing through a 5 m long single-mode fiber with $\beta_2 = -21 \times 10^{-3}\ \text{ps}^2/\text{m}$ (higher-order GVD and nonlinearity are ignored). The broadened pulse's duration is 676 fs. We also measured the pulse duration for 10, 15, and 20 m long fibers, the extracted pulse durations being 1.343, 2.105, and 2.913 ps, respectively. These measurements are necessary for the calibration of the spatial-spectrum interferometry (SSI) that is used to reconstruct pulse in a single-shot manner.

Supplementary Note 2: The double hologram pulse (D-shaper) shaper system

A simplified schematic of the D-shaper system's section is displayed in Supplementary Figure 2(a). The input laser pulse coming from the OPO system, $E_{OPO}(t)$, is first dispersed into its spectral components by a diffraction grating, and then use a lens to focus the diffraction beam. At the Fourier plane of the input lens, the electric field is transformed from the spectral-temporal

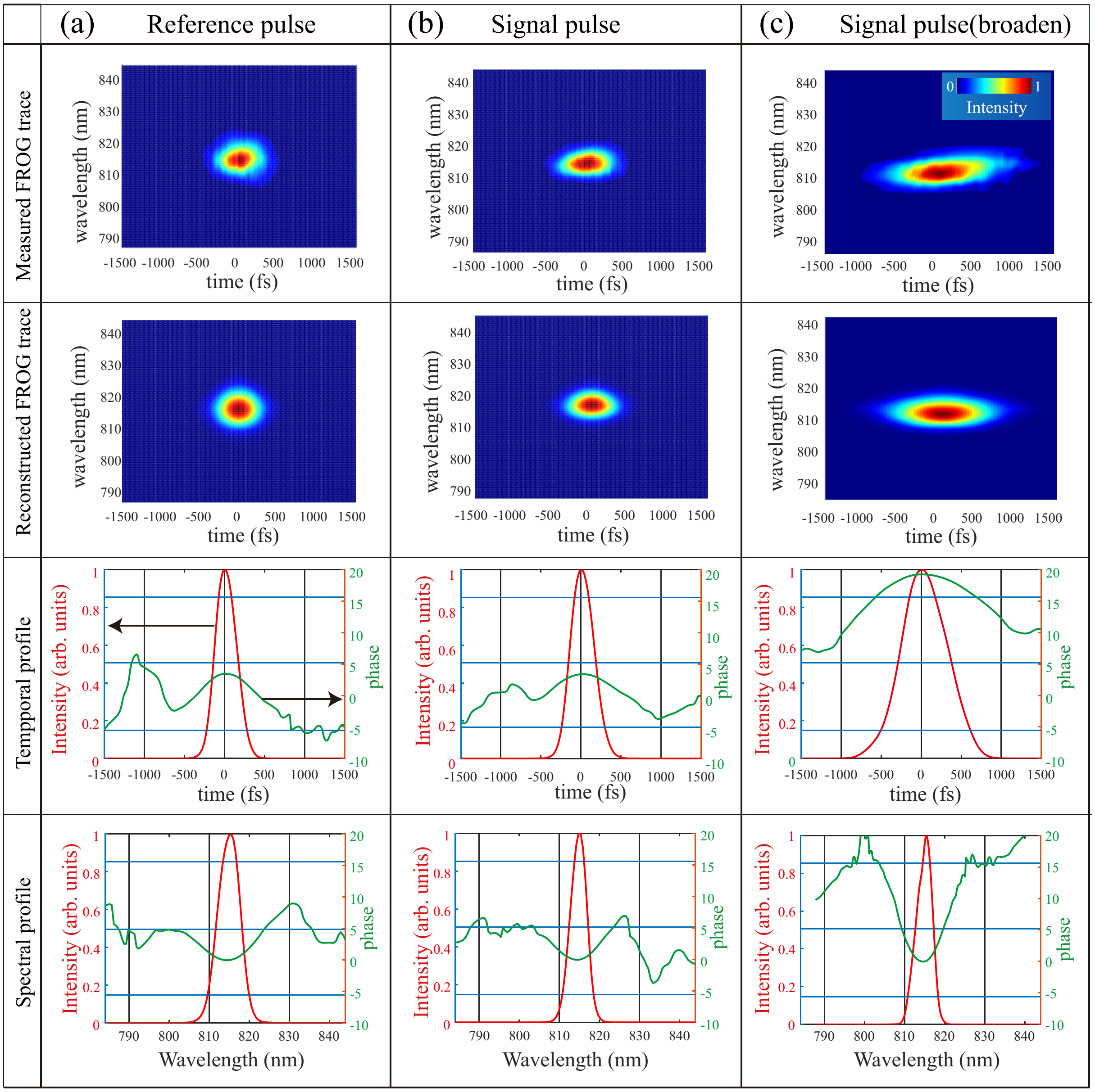

Supplementary Figure 1. **The reconstruction of the pulses using the collinear FROG system.** Columns (a), (b), and (c) represent the reference, signal, and a broadened signal pulses, respectively. Shown in each column, from top to bottom, are the measured and reconstructed FROG trace, intensity, and phase in the temporal and spectral domains, respectively. The color-coding in these panels represents the relative intensity, which is normalized to take values uniformly from minimum to maximum.

domain into the spatial-temporal one, written as $E_{Initial}(x_\omega)$, where x_ω is the horizontal spatial position. We put the first hologram (the top half of the SLM) at the Fourier plane and load the desirable spectral phase. Based on the spectral-spatial dispersion relationship, one can obtain the frequency resolution at the Fourier plane (the upper part of the SLM in Supplementary Figure

2(a)) as [3, 4]

$$\frac{d\omega}{dx} = \frac{2\pi c d \cos(\theta_d)}{\lambda_0^2 f_0}. \tag{2}$$

Here, c is the light speed in vacuum, d is the spatial period of the grating, and θ_d is its first-order diffraction angle. Due to the diffraction, each spectral component acquires a finite size δx in the Fourier plane [3],

$$\delta x = \sqrt{2\ln(2)}\frac{\cos(\theta_i) f_0 \lambda_0}{\cos(\theta_d)\pi w_{\text{in}}}. \tag{3}$$

Here, f_0 is the focus length of the lens, w_{in} is the waist of the input beam, while $\theta_{i,d}$ are the input and first-order diffraction angles of the optical grating. Using Supplementary Equation (2) and (3), one obtains the temporal window for the D-shaper system,

$$T_w = (4\ln 2)/\delta_w. \tag{4}$$

Using a $4f$ or $2f_1$-$2f_2$ imaging system, the field on the first hologram (the upper half of the SLM) is imaged onto the second hologram (the bottom half of the SLM). On the second hologram, we add the spectral phase necessary to realize the regular or fractional GVD, *viz.*,

$$\phi_{\text{SC}} = -\left(\sum_{k=2,3,\ldots}\frac{\beta_k}{k!}\omega^k + \frac{D}{2}|\omega|^\alpha\right)L, \tag{5}$$

where β_k is the k-th regular GVD coefficient, D the fractional GVD coefficient, α is the LI, and L the propagation length. Between the two holograms, in the focus of the first lens, an aperture is used to select only the first diffraction order [5, 6]. For our system, the input beam size w_{in} is about $1\,\text{mm}$; the input and first-order diffraction angles are $51.44°$ and $25°$ respectively, for the optical grating (Thorlabs GR13-1208, $1200/\text{mm}$, blaze wavelength $750\,\text{nm}$, $12.7 \times 12.7 \times 6\,\text{mm}$). We have measured the spatial dispersion of about $1.82\,\text{nm/mm}$ when using a double-slit for the calibration [7]. The expected spatial dispersion is calculated to be $\approx 1.88\,\text{nm/mm}$. The expected temporal window is calculated to be $\approx 8.78\,\text{ps}$. By moving the relative delay (R4 in Figure 5 of the main text), the measured temporal window is $\approx 8\,\text{ps}$. This value determines the upper limit for the propagation length in the FSE. Supplementary Figure 2(b1)-(e1) shows the examples of the holograms used in the D-shaper, and their respective interference fringes, recorded by the CCD of the SSI system (Supplementary Figure 2(b2)-(e2)). In the hologram, we have added an additional spatial optical grating phase (along the y direction) to generate the first-order diffraction, which allows us to avoid the crosstalk and then improve the signal to noise [5–7].

There are several advantages of using a D-shaper regime with the double hologram. On the one hand, two holograms can control the initial profile and propagation independently. On the other hand, it is possible to improve the frequency resolution and the temporal windows of the second hologram, by changing the imaging lens in Supplementary Figure 2(a), which would extend the limitation in propagation length.

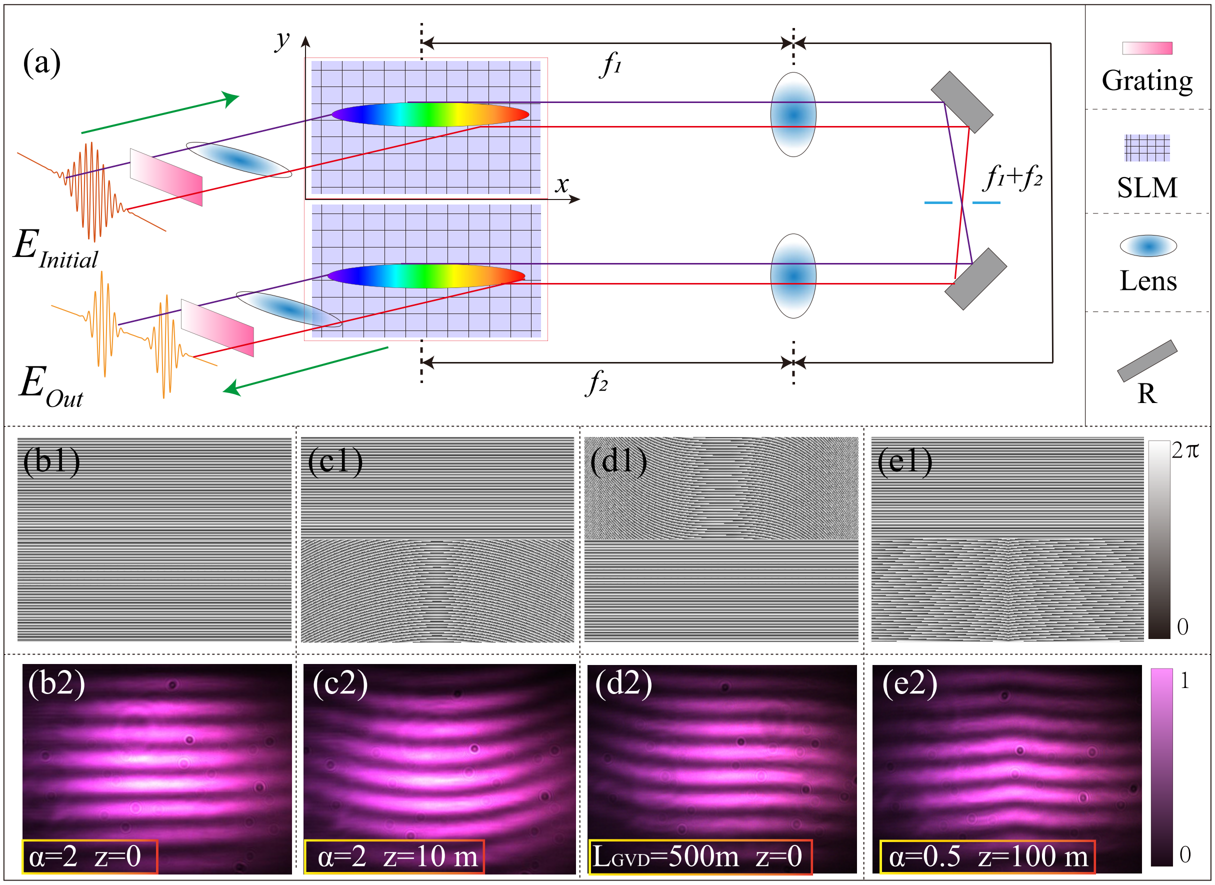

Supplementary Figure 2. **A schematic of the double hologram pulse-shaper system (D-shaper) and interferometric fringes recorded by SSI.** (a): The main parts of the D-shaper, including the SLM, lens, and aperture. (b) Interference fringes in the SSI for a flat phase in both the initial condition and the propagation segment. (c) The flat-phase initial condition, and the second-order spectral phase are equivalent to the propagation through a 10 m long single-mode fiber. (d) The third-order spectral phase produced by $L_{\mathrm{GVD}} =$ 500 m, as the initial condition which generates a temporal Airy pulse, and a flat phase for propagation. (e) The flat-phase initial condition and fractional phase for LI $\alpha = 0.5$ and the propagation length of 100 m. The color-coding in these panels represents the relative intensity, which is normalized to take values uniformly from minimum to maximum.

Supplementary Note 3: The single-shot spatial-spectrum interferometry

In the spatial-spectrum interferometry (SSI), the reference and signal pulse is made slightly non-collinear at the BS (beam splitter), so that they overlap and interfere in the Fourier plane (CCD camera) after passing through the diffraction grating, where the cylindrical lens is used to form a two-dimensional spatial-spectral pattern. Then, using the Fourier filtering algorithm [8, 9], one can reconstruct the spectral phase $\phi_r(\omega)$. The actual spectral phase of the signal pulse can

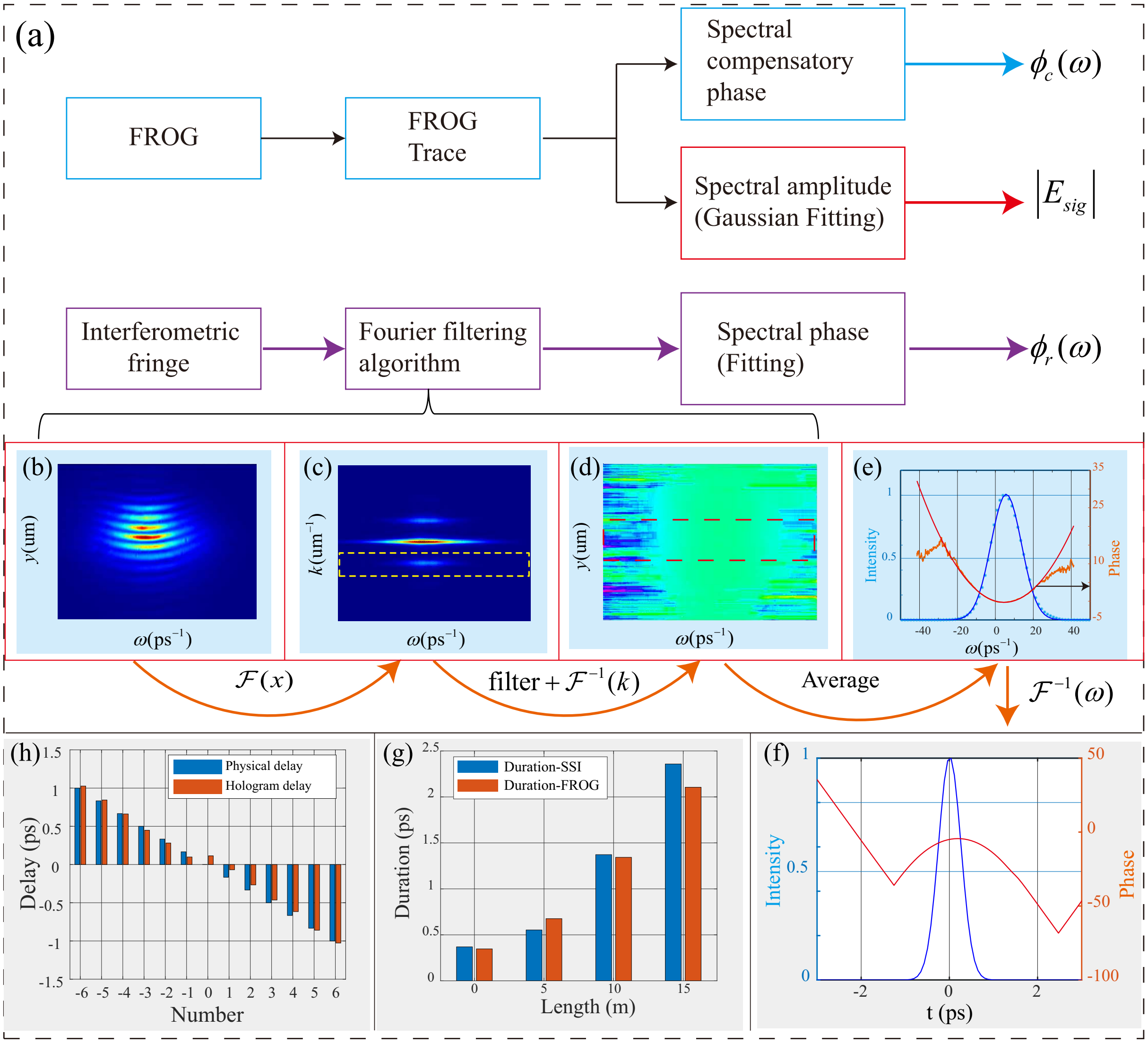

Supplementary Figure 3. **The procedure for the spectral phase reconstruction by means of the spatial spectrum interferometry(SSI).** (a) the reconstruction procedures for the SSI, where the Fourier filtering algorithm is divided into (b) the recorded interference pattern on CCD, (c) the results produced by the Fourier transform along the vertical axis, (d) the extracted phase after the application of the filtering and inverse Fourier transform. (e,d) the reconstructed intensity and phase in the spectral (e) and temporal (f) domains. (g) the pulse durations as produced by the SSI and FROG (blue and orange bars, respectively) for different values of the second-order phase. (h) the relative optical delay (first-order spectral phase) between the reference and signal pulses, as extracted from the SSI (orange bars). Here the blue bars represent the optical delay produced by element R4 in the setup presented in Figure 5 of the main text. Orange bars in (g) represent the cases when the first-order spectral phase is generated by the hologram.

be obtained by adding phase $\phi_c(\omega)$ of the reference pulse measured by the FROG. To ensure the accuracy of the SSI, we compare the reconstruction of the pulse durations with the results produced by the FROG system (Supplementary Figure 1). These results are shown in the next, which also includes details of the reconstruction.

Supplementary Figure 3 illustrates the reconstruction procedure used by the spatial-spectrum interferometry (SSI). In the Fourier plane of the cylindrical lens, the signal, and reference field could be roughly defined as $|E_{\text{sig}}(x_\omega, y)| \exp\left(i\phi_{\text{sig}}(x_\omega)\right)$ and $|E_{\text{ref}}(x_\omega, y)| \exp\left(i\phi_{\text{ref}}(x_\omega)\right)$, respectively. At the surface of the CCD camera, the theoretical two-dimensional interference pattern $I(x_\omega, y)$ can be written as [10]

$$I(x_\omega, y) = A^2(y) \begin{bmatrix} E_{\text{ref}}^2(x_\omega) + E_{\text{sig}}^2(x_\omega) + 2E_{\text{ref}}(x_\omega)E_{\text{sig}}(x_\omega) \\ \times \cos\left(\phi_{\text{sig}}(x_\omega) - \phi_{\text{ref}}(x_\omega) - 2K(x_\omega) y \sin(\theta)\right) \end{bmatrix}, \tag{6}$$

where x_ω is the spatial horizontal position, which is proportional to ω, and y is the spatial vertical coordinate. $K(x_\omega)$ is equal to $2\pi/\lambda$, where λ is the wavelength corresponding to the position x_ω, and θ is the angle between the reference and signal beam at the CCD. The interference pattern disappears at $\theta = 0$. We set the y component of the reference and signal to be the same as $A(y)$. To obtain the phase of ϕ_{sig}, we apply the Fourier filtering algorithm to the recorded interference patterns [8, 9], which includes four steps (Supplementary Figure 3(b)-(e))—the Fourier transform along the y axis, the filter, and the inverse Fourier transform—and averaging and fitting the reconstructed phase. By performing the Fourier transform for the interference pattern (Supplementary Equation (6)) along the y axis, we obtain the following expression:

$$\mathcal{F}_y\left[I(x_\omega, y)\right] = \underbrace{\left(E_{\text{ref}}^2(x_\omega) + E_{\text{sig}}^2(x_\omega)\right) \mathcal{F}_y\left[A(y)^2\right]}_{\text{DC term}} + 2E_{\text{ref}}(x_\omega)E_{\text{sig}}(x_\omega) \times \left(\begin{array}{l} \underbrace{\mathcal{F}_y\left[\frac{1}{2}\exp\left(i\left(\phi_{\text{sig}}(x_\omega) - \phi_{\text{ref}}(x_\omega) - 2K(x_\omega) y \sin(\theta)\right)\right) A(y)^2\right]}_{\text{upper band}} \\ + \underbrace{\mathcal{F}_y\left[\frac{1}{2}\exp\left(-i\left(\phi_{\text{sig}}(x_\omega) - \phi_{\text{ref}}(x_\omega) - 2K(x_\omega) y \sin(\theta)\right)\right) A(y)^2\right]}_{\text{lower band}} \end{array} \right). \tag{7}$$

Here, three parts appear, *viz.*, the DC term and upper and lower bands near the DC term, which can be found as shown in Supplementary Figure 3(c). By adding a rectangular filter, we retain only a single band—e.g., the lower band in the yellow box of Supplementary Figure 3(c). In this case, the remaining part is:

$$2E_{\text{ref}}(x_\omega)E_{\text{sig}}(x_\omega) \times \mathcal{F}_y\left[\frac{1}{2}\exp\left(-i\left(\phi_{\text{sig}}(x_\omega) - \phi_{\text{ref}}(x_\omega) - 2K(x_\omega) y \sin(\theta)\right)\right) A(y)^2\right]. \tag{8}$$

Then, we perform the inverse Fourier transform and produce the new field, E_R:

$$E_R = E_{\text{ref}}(x_\omega)E_{\text{sig}}(x_\omega)A(y)^2 \times \exp\left(-i\left(\phi_{\text{sig}}(x_\omega) - \phi_{\text{ref}}(x_\omega) - 2K(x_\omega) y \sin(\theta)\right)\right). \tag{9}$$

Here, θ can be inferred with the help of the fringe spacing [9], therefore the spatial-phase term $2K(x_\omega)y\sin(\theta)$ can be calculated. The phase reference term $\phi_{\rm ref}(x_\omega)$ has been determined employing the FROG system (see Supplementary Figure 3(a)). The term $\phi_{\rm ref}(x_\omega)$ remains constant in the course of the experiments, and the manipulations were performed by the shaper and filter solely with the signal beam. Supplementary Figure 3(d) is the phase distribution produced by removing the spatial phase term (adding the spatial optical grating). By averaging the phase along the y axis in the special range, i.e., in the red box in Supplementary Figure 3(d), one can retrieve the one-dimensional phase distribution.

There is one big disadvantage of this regime of SSI: the reconstructing results are strongly affected by spatial interference patterns recorded by CCD. To get rid of the fluctuations due to spots in the fringe (sometimes, they are unavoidable, such as holes in images of Supplementary Figure 2(b2)-(e2)), we fit the phase to the power series, i.e., $p_0 + p_1\omega^1 + p_2\omega^2 + p_3\omega^3 + ...$for the integral spectral phase. By addressing the spectral amplitude and phase from the reference $\phi_{\rm ref}$, which have been produced by the FROG system, we obtain the final field distributions in the spectral and temporal domains.

Supplementary Figure 3(b) shows the fringes when the hologram introduces the second-order phase (with $L = 5$ m). The result of the Fourier transform along the y axis is shown in Supplementary Figure 3(c), where three bands are clearly seen, and we use a spatial filter (the yellow box) and then perform the inverse of the Fourier transform. The inverted spatial optical phase and the unwrapped phase are shown in Supplementary Figure 3(d). Supplementary Figures 3(e) and (f) are the reconstructed intensity and phase profiles in the spectral and temporal domains, respectively. An obvious second-order phase appears at the center of the spectral intensity, where the intensity is the fitting curve that uses a Gaussian function for the blue-star data provided by the FROG reconstruction (Supplementary Figure 1(b)).

Because several BSs and PBSs are arranged to control the beam's propagation, a second-order phase chirp is added to the signal and reference pulses, as can be seen from the FROG reconstruction (Supplementary Figure 1). Based on the obtained phase, the chirp is ≈ 0.0066 ps^2, which corresponds to a fiber ($|\beta_2| = 21 \times 10^{-3}$ps^2/m) with length ≈ 0.634 m. Taking into account the chirp in phase $\phi_{\rm ref}$, the results obtained from the SSI are close to the FROG counterparts. In the experiments, we have made several reconstructions. In particular, Supplementary Figure 3(g) corresponds to the second-order phase with the fiber lengths $0, 5, 10$, and 15 m, respectively. The pulse durations are $[0.347, 0.676, 1.343, 2.105]$ ps from the FROG reconstructions, and $[0.370, 0.553, 1.372, 2.36]$ ps from the SSI. Also, in Supplementary Figure 3(h) we display the situation for the input pulse with the first-order phase. To this end, we move the mirror R4 fixed on one translation stage in the setup (section SSI in Supplementary Figure 5). Limited to the spatial resolution of the translation stage, the minimum difference between two adjacent number/times delays is set to ≈ 166.65 fs. The measured results of time delay are shown in Supplementary

Figure 3(h), where the blue bars marked as “physical delay” are the results of the reconstruction by scanning R4; the orange bars represent the corresponding results obtained when the time delay is created by a series of the hologram with the first-order spectral phase, therefore they are labeled as “hologram delay”. Values of the temporal delay produced by the two methods are similar. These results show that the single-shot SSI, built into our setup, is reliable, producing robust reconstructions.

The methods of SSI (spatial-spectrum interferometry) have been applied in other experiments, such as real-time measurements or the work with the material’s dispersion [10, 11]. In our setup, the SSI is applied with some modifications. Firstly, it is the use of the spectral amplitude as measured by the FROG system, instead of that directly reconstructed from SSI. If the amplitude is directly extracted from the SSI reconstruction, some distortions appear in the amplitude curve. Secondly, we make the spatial tilt angle θ adaptive, according to the interference pattern. Based on Supplementary Equation (6), the angle θ between the reference and signal pulses should be fixed. Actually, it is slightly varying at different spots of the interference pattern due to the imperfection in experiments. The adaptive strategy could remove the slight spatial-phase jitter in the course of the reconstruction. Thirdly, we employ the fitting model to smooth the reconstructed phase (see Supplementary Figure 3(e)), so as to remove fluctuations in the course of the detection due to the flecks on the surface of CCD. Also, it could partly overcome the spectral resolution and sampling issues in the framework of the SSI [12].

---

[1] Rick Trebino, Kenneth W DeLong, David N Fittinghoff, John N Sweetser, Marco A Krumbügel, Bruce A Richman, and Daniel J Kane. Measuring ultrashort laser pulses in the time-frequency domain using frequency-resolved optical gating. *Rev. Sci. Instrum.*, 68(9):3277–3295, 1997.

[2] Shilong Liu, Yudong Cui, Zhiyuan Zhou, and Ebrahim Karimi. An efficient collinear frog system to character the ultrafast infrared laser pulse. In *2021 Photonics & Electromagnetics Research Symposium (PIERS)*, pages 2690–2694. IEEE, 2021.

[3] Antoine Monmayrant, Sébastien J Weber, and Béatrice Chatel. A newcomers guide to ultrashort pulse shaping and characterization. *J. Phys. B*, 43(10):103001, 2010.

[4] Andrew M Weiner. Ultrafast optical pulse shaping: A tutorial review. *Opt. Commun.*, 284(15):3669–3692, 2011.

[5] Eliot Bolduc, Nicolas Bent, Enrico Santamato, Ebrahim Karimi, and Robert W Boyd. Exact solution to simultaneous intensity and phase encryption with a single phase-only hologram. *Opt. Lett.*, 38(18):3546–3549, 2013.

[6] Shi-Long Liu, Qiang Zhou, Shi-Kai Liu, Yan Li, Yin-Hai Li, Zhi-Yuan Zhou, Guang-Can Guo, and Bao-Sen Shi. Classical analogy of a cat state using vortex light. *Commun. Phys.*, 2(1):1–9, 2019.

[7] Shilong Liu, Yudong Cui, Ebrahim Karimi, and Boris A Malomed. On-demand harnessing of photonic soliton molecules. *Optica*, 9(2):240–250, 2022.

[8] Ian A Walmsley and Christophe Dorrer. Characterization of ultrashort electromagnetic pulses. *Adv. Opt. Photonics*, 1(2):308–437, 2009.

[9] Selcuk Akturk, Xun Gu, Pamela Bowlan, and Rick Trebino. Spatio-temporal couplings in ultrashort laser pulses. *J. Opt.*, 12(9):093001, 2010.

[10] Takasumi Tanabe, Hiroshi Tanabe, Yuichi Teramura, and Fumihiko Kannari. Spatiotemporal measurements based on spatial spectral interferometry for ultrashort optical pulses shaped by a fourier pulse shaper. *J. Opt. Soc. Am. B*, 19(11):2795–2802, 2002.

[11] D Meshulach, D Yelin, and Yaron Silberberg. Real-time spatial–spectral interference measurements of ultrashort optical pulses. *J. Opt. Soc. Am. B*, 14(8):2095–2098, 1997.

[12] Christophe Dorrer, Nadia Belabas, Jean-Pierre Likforman, and Manuel Joffre. Spectral resolution and sampling issues in fourier-transform spectral interferometry. *J. Opt. Soc. Am. B*, 17(10):1795–1802, 2000.
