# Supplementary material for: Experimental realisations of the fractional Schrödinger equation in the temporal domain
Source: Nat Commun. 2023 Jan 14;14:222. doi: 10.1038/s41467-023-35892-8 (PMC9840624; doi:10.1038/s41467-023-35892-8)
Supplement: Supplementary file 3 — Description of Additional Supplementary Files [file 41467_2023_35892_MOESM3_ESM.pdf]

Description of Additional Supplementary Files

Supplementary Movie 1: The variation of Levy index α in the fractional Schrödinger equation.

Simulations of the dynamics governed by the fractional Schrödinger equation with values of the Levy index α from 0 to 2. The horizontal axis represents time extending from -5 ps to 5 ps; the vertical axis is the propagation distance running from 0 to 100 m.

Supplementary Movie 2: The dynamics for the temporal Airy wave governed by the fractional Schrödinger equation.

Simulations of the pulse dynamics for an input profile with a third-order spectral phase. The first panel shows the temporal-pulse dynamics for α= 0.5, 1, 1.25, 1.5, 1.8, and 2. The second panel is the extracted trajectory of the main lobe of the Airy pulse. The third panel shows the relation between the fitting parameter g and α. The last panel shows the relation between g and α on the logarithmic scale.

Supplementary Movie 3: The pulse with the fractional phase governed by the regular Schrödinger equation: "fractional-phase protection" effect.

Simulations and experimental results for the dynamics of pulses carrying a fractional phase propagating in a regular dispersion material, where α takes value 0.4, 0.6, 0.8, and 1. The first and second rows show, respectively, the results of the simulations and experimental measurements. The third row displays the extracted fractional and regular (second-order) phase shifts, respectively.
