# Supplementary figures and images for: Experimental realisations of the fractional Schrödinger equation in the temporal domain
Source: Nat Commun. 2023 Jan 14;14:222. doi: 10.1038/s41467-023-35892-8 (PMC9840624; doi:10.1038/s41467-023-35892-8)

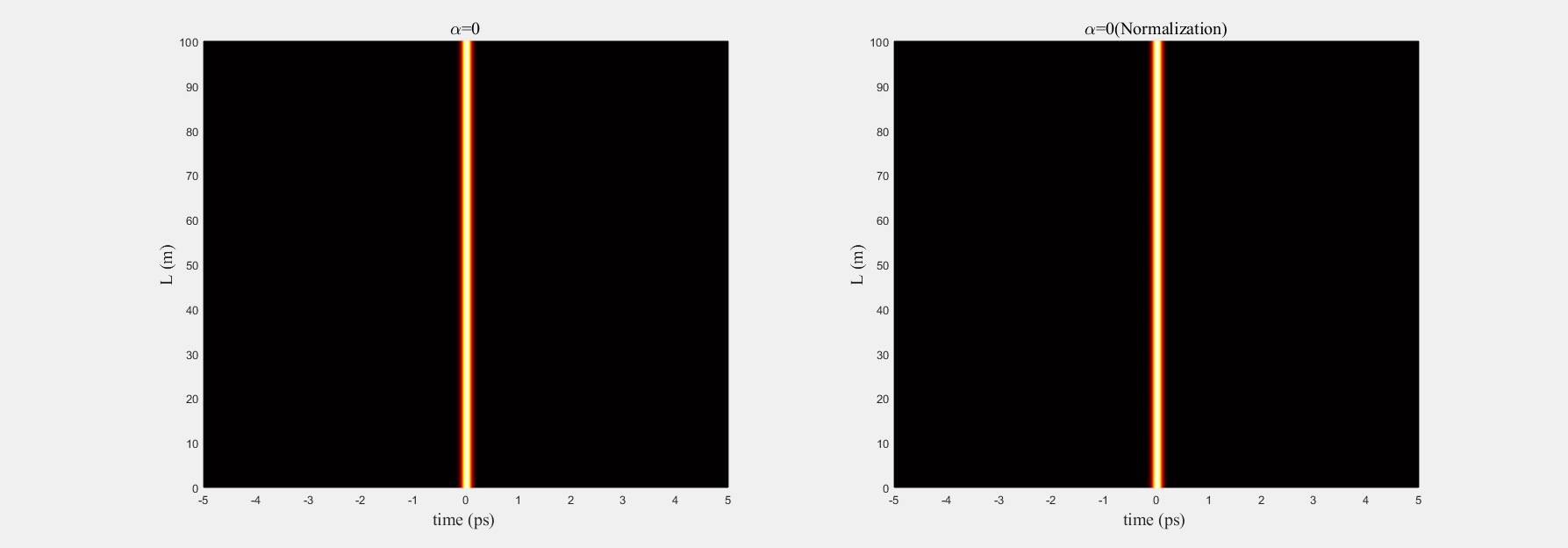

Supplement: Supplementary file 4 — Supplementary Movie 1 [file 41467_2023_35892_MOESM4_ESM.gif]

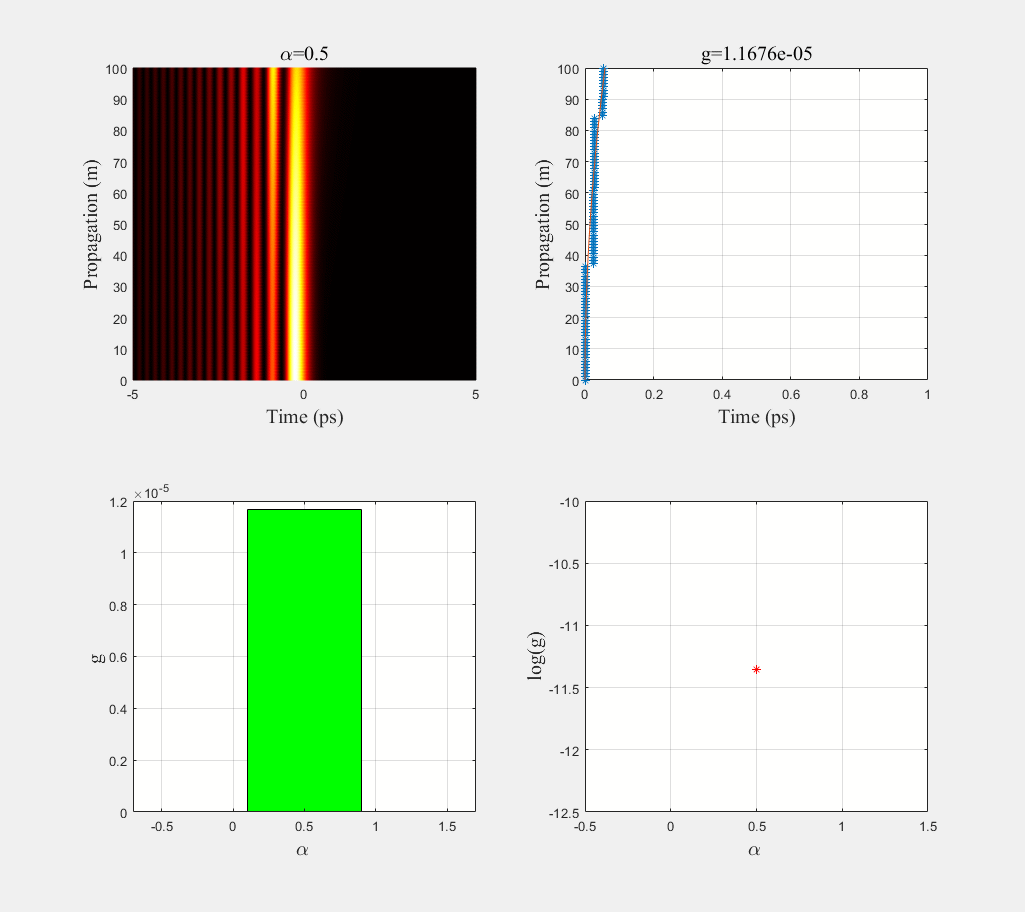

Supplement: Supplementary file 5 — Supplementary Movie 2 [file 41467_2023_35892_MOESM5_ESM.gif]

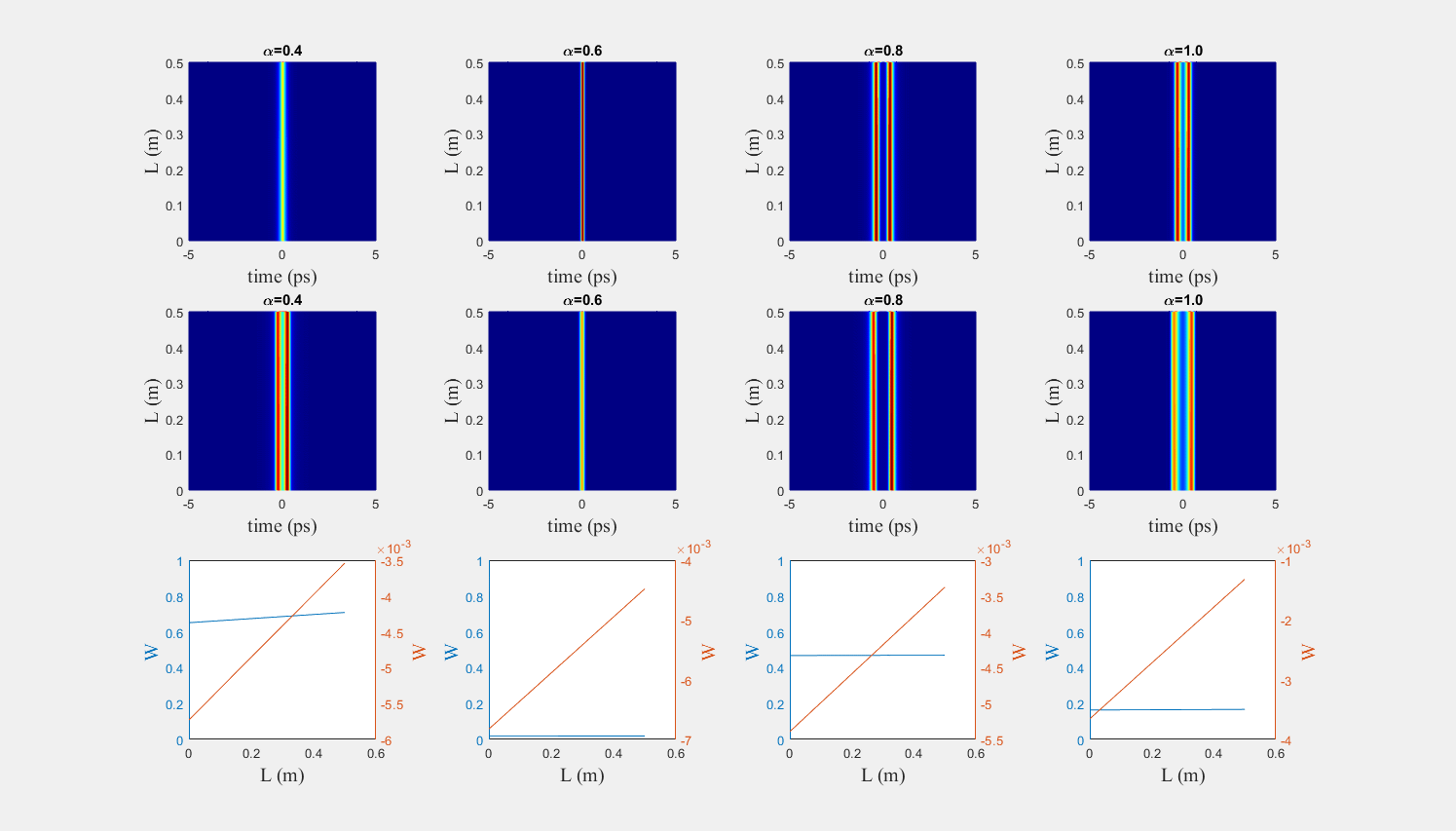

Supplement: Supplementary file 6 — Supplementary Movie 3 [file 41467_2023_35892_MOESM6_ESM.gif]
